# All inside full thickness quadriceps tendon ACL reconstruction: Long term follow up results

**DOI:** 10.1186/s40634-020-00226-w

**Published:** 2020-03-14

**Authors:** Hernan Galan, Mateo Escalante, Franco Della Vedova, Daniel Slullitel

**Affiliations:** Instituto “Dr Jaime Slullitel”, Rosario - Santa Fe, Argentina

**Keywords:** ACL reconstruction, Quad tendon, All inside

## Abstract

**Purpose:**

The aim of this study is to evaluate results of anterior cruciate ligament reconstruction (ACL) using an All-Inside Full Thickness Quadriceps Reconstruction technique at 5 years follow up.

**Methods:**

This is a Retrospective cohort study of patients undergoing ACL reconstruction. Inclusion criteria for this report were isolated primary ACL reconstructions without chondral lesions (Grade III/IV Outerbridge), using autologous full-thickness quadriceps tendon (FQT) graft with bone block, with an “all-inside” technique. Functional scales of Lysholm, IKDC, Tegner and objective results of side to side difference (KT1000) were used for this evaluation. Additionally, complications and comorbidities were also analyzed.

**Results:**

Two hundred and ninety-one ACL reconstructions were retrospectively reviewed at 5 years postoperatively; 268 (92.1%) were men and 23 (7.90%) women. Lysholm Score improved from 64 (SD = 6.09) to 91 (SD = 6.05) points average. IKDC showed 59.79%, excellent and 3.4% good results. Arthrometric analysis showed that 259 knees (89%) had a difference of less than 3 mm. Median pre-injury Tegner score was 9 (Range 4–10), while final median Tegner activity level at 5 years was 8 (Range 4–10). Among comorbidities, 5.15% of the patients presented anterior knee pain. No visualization difficulties or significant hematomas were found.

**Conclusion:**

Use of all inside FQT for ACL reconstruction in a young, high demand sports population, present at 5 years, good to excellent results, functionally and objectively, with low rates of complications and comorbidities.

## Purpose

Optimal ACL Reconstruction needs a strong ACL Graft with minimum site morbidity. Bone-patellar tendon bone graft (BPTB) is widely used in reconstruction among athletes for its mechanical resistance. However, anterior knee pain, donor-site morbidity, and/or knee flexion contracture, are reported problems following surgery [33, 35, 39].

Another commonly adopted choice, as reported in a 2013 survey by the American Academy of Orthopedic Surgeons, [1] is hamstring grafts (HT), which is used by 44% of surgeons on primary ACL reconstruction, in adult recreational athletes. However, there might be complications related to HT harvest, including saphenous nerve injuries, tendon amputation during graft harvest, and presence of smaller graft diameters increasing the risk of re-rupture [8, 29]. Quadriceps tendon (QT) graft was introduced in 1979 by Marshall et al. [30], but its modern use begins with Blauth description [5]. In 1999, Fulkerson [10] described its use without a bone block. A recently published systematic review revealed that quadriceps tendon provides knee stability, functional scores, rupture rates comparable to the BPTB and hamstring tendon grafts, but less anterior knee pain rates than BPTB, and better flexor strength than HT [40].

An additional advantage of QT is a reliable graft size (as is the case with BPTB) therefore, surgeon can choose graft width at harvesting [40]. Also, collagen fiber thickness of QT is larger, [14] thereby leaving a thicker intraarticular ACL. Collagen percentage is higher, increasing its resistance to rupture [37]. Use of QT reconstruction has recently increased with advanced graft harvesting techniques. In 2014, Middleton et al. [31] reported that 11% of surgeons in 20 countries preferred to use quadriceps tendons in their surgeries.

ACL reconstructions with partial- or full-thickness quadriceps tendons have been described in the world literature. Previous anatomical analyses of the quadriceps tendon have revealed that the average thickness of the distal tendon is approximately 8 mm with an average thickness of 16–18 mm at the patellar insertion site [40, 43]. Theoretical advantages of a full-thickness quadriceps tendon include increased graft tensile strength, lower rates of graft failure and improved stability, while theoretical drawbacks include increased donor-site morbidity as well as injuring the knee joint capsule or suprapatellar bursa. As evidence continues increasing in favor of the use of the quadriceps tendon [4, 16, 34, 38] and as its use is growing in popularity, [40] it will become increasingly important to optimize techniques for reconstructing the ACL with it.

Our aim is to report long term results of an “all inside” FQT [41] tendon graft, in a high demand population. Our primary hypothesis is that All inside FQT ACL Reconstruction is a suitable procedure in high demand sports patients, with low morbidity rates.

## Methods

A retrospective analysis of patients undergoing ACL reconstruction was carried out and a search of our database between January 2009 and December 2013 was conducted.

Inclusion criteria for this report were isolated primary ACL reconstructions without chondral lesions (Grade III/IV Outerbridge), using an autologous full-thickness quadriceps tendon graft with bone block, with an “all-inside” technique.

## Evaluation of outcomes

All patients completed a standardized, validated outcome questionnaire (filled by themselves), developed by the IKDC and the Lysholm score, preop and at 5 years after surgery. Patients return to sports activity and level was also assessed using Tegner’s Score. Anterior knee displacement was measured mechanically with the KT-1000 [34] and results were compared with healthy, opposite knee. Measurements were taken with knee in 25 degrees of flexion and maximum manual force. Anterior knee pain was evaluated with ability to walk on knees [18]. All returning patients were assessed by a non independent observer.

## Surgical technique

**Fig. 1:**
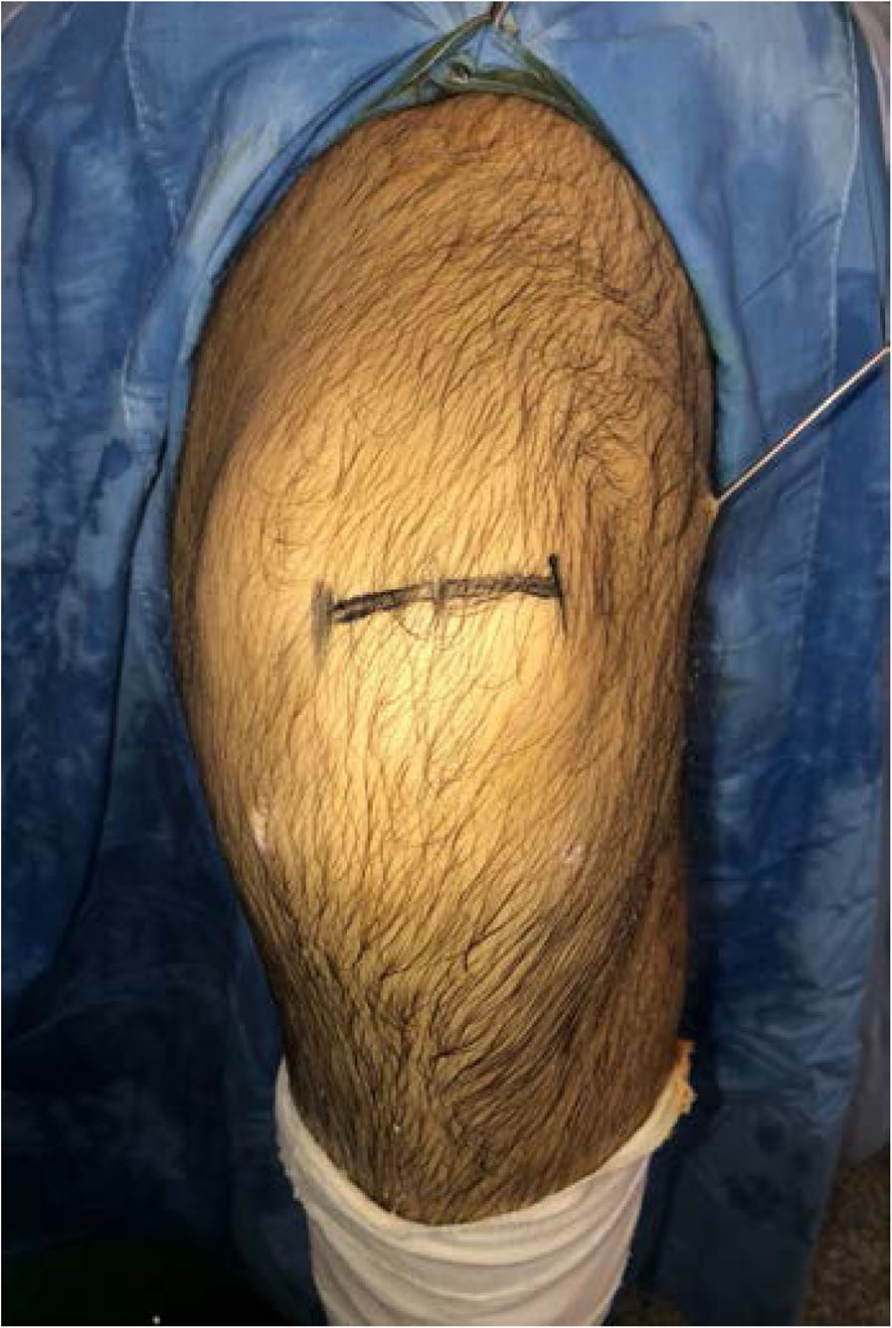
Horizontal Skin Incision

**Fig. 2:**
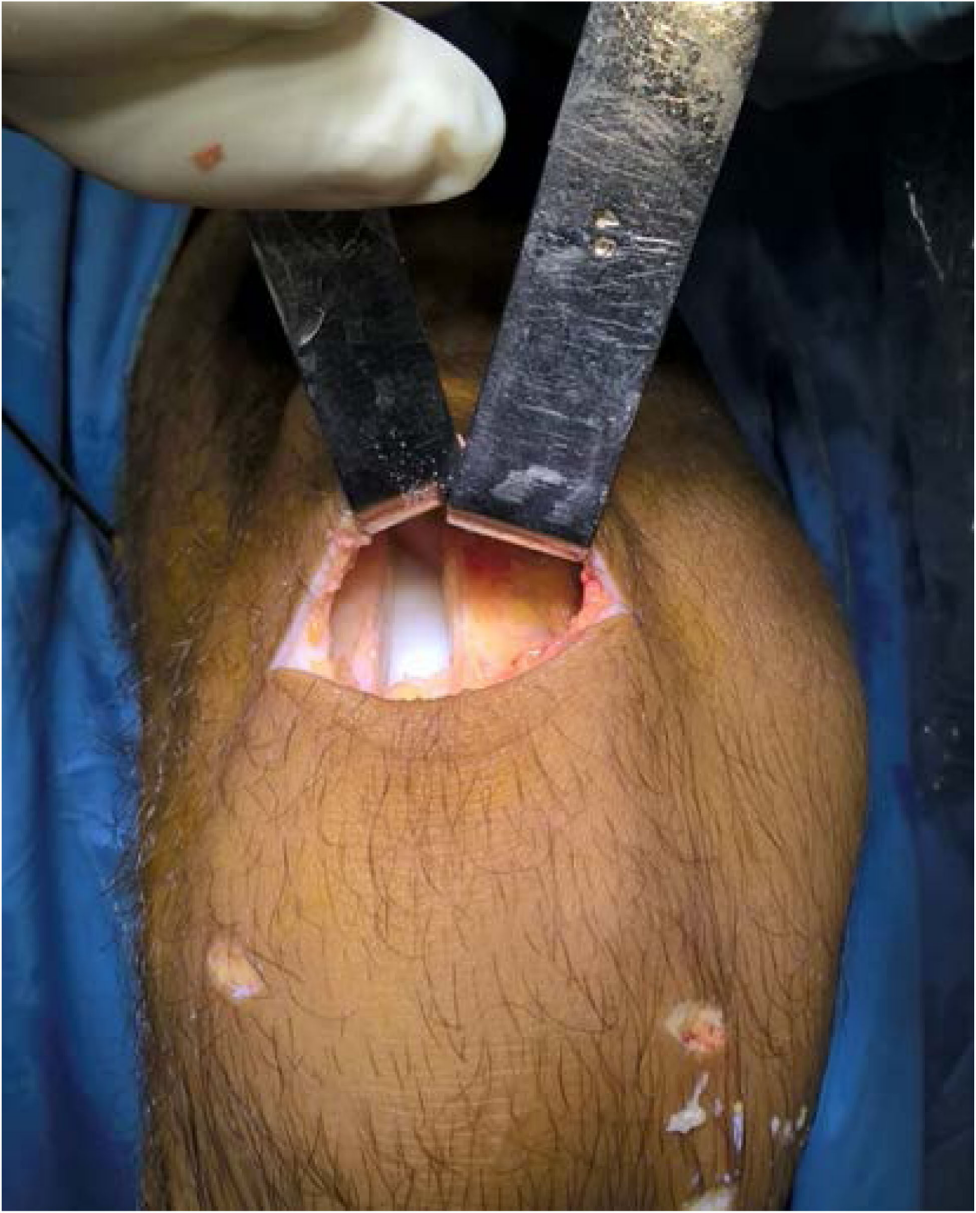
Open Suprapatelar Pouch after graft harvesting

**Fig. 3:**
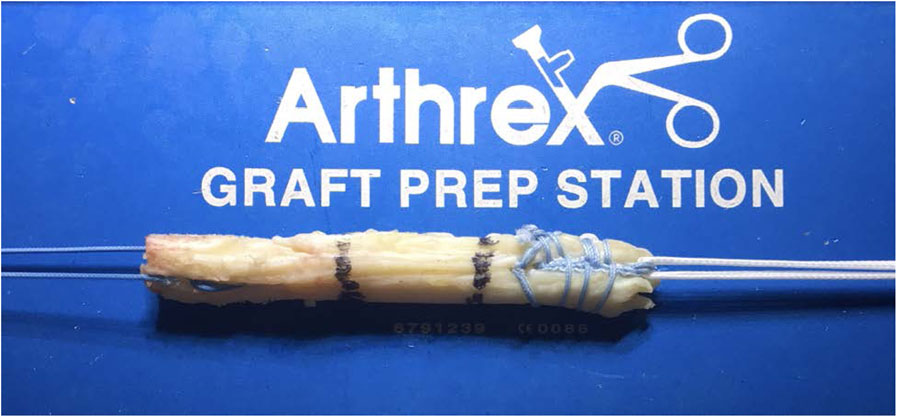
Graft Preparation (Krakow Suture in the Tendon Part)

**Fig. 4:**
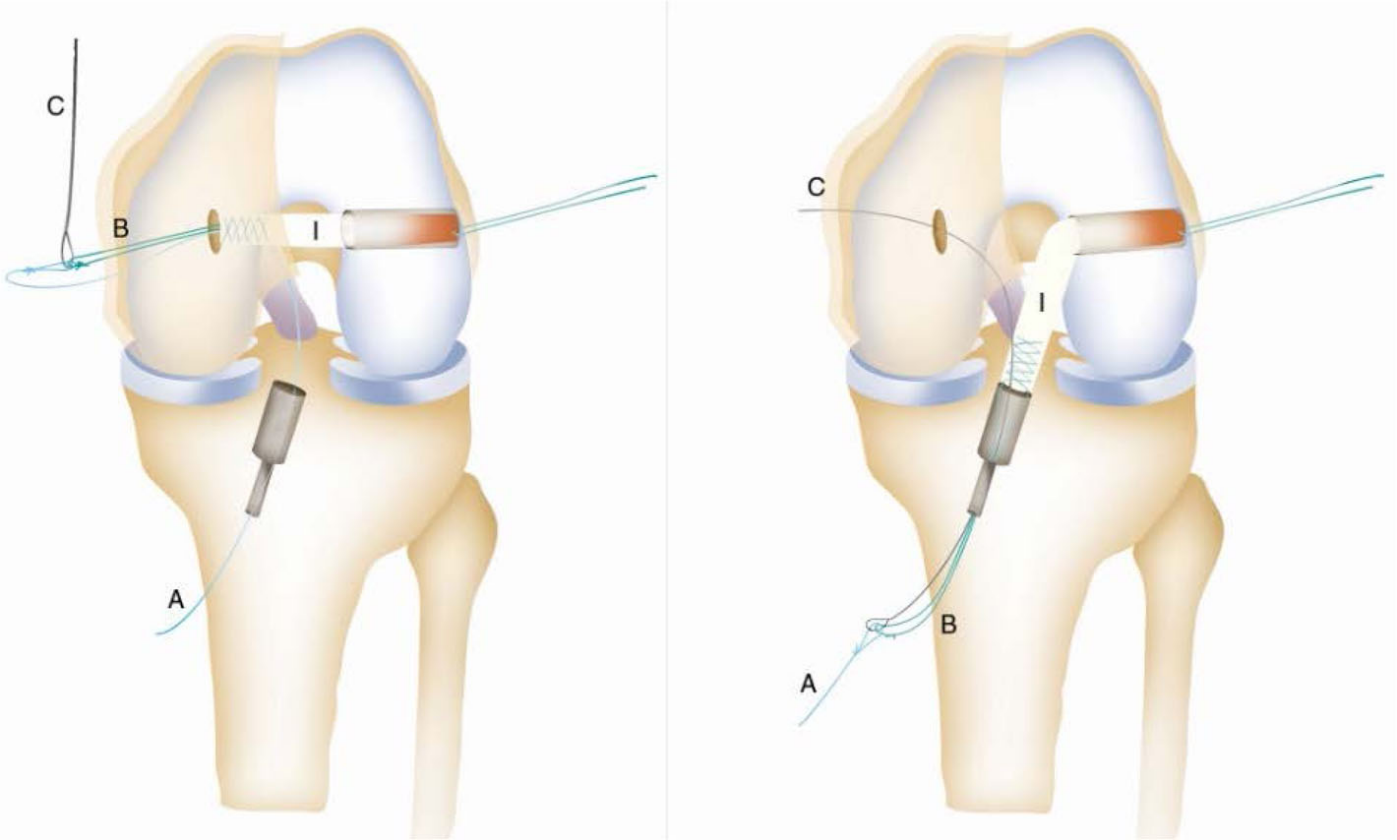
Graft Passage. 1- Tendon Side on Femoral Tunnel, leading suture on tibial side. 2- By pulling on the leading suture the graft slides on the tibial side. A. Leading suture; B Krakow suture; C Nitinol eyeled wire; I QT Graft

Patient with regional block, lying supine with a circumferential knee holder and well leg abducted, bed foot dropped, knee flexed 90 degrees. Standard anterolateral and anteromedial portal are made. After performing a diagnostic arthroscopy, the intercondylar notch is cleaned. Through an additional low anteromedial portal in 110 knee flexion, we aim a 6 mm offset guide (Arthrex), in ACL footprint (as described by O’Donnel) [32], to drill a 10 mm hole or more and host a same diameter QT graft. This hole should go as close as possible to the femoral lateral cortex to have enough room to permit graft sliding without losing tension, as this is an all inside technique. After that, by a horizontal incision on the upper patellar pole (Fig. 1), by blunt dissection, we search vastus medialis lateral side, and perform a vertical incision 3 mm lateral to it, 6 cm long approx. (as this is the thickest QT zone) (6) (Fig. 2), then finish extracting a 10 mm or more tendon width, full thickness. Besides, a 15 mm long upper patellar bone block is taken with saw, previously drilling a small hole to host a fiberwire for guiding and tensioning purposes. No special measures are taken to prevent joint opening while raising the graft. (Photo 2) If this happens, we find useful to flex the knee as it tends to stop fluid leakage or, to perform Arthroscopy without fluid. With these tricks, no major visualization issues were found in our whole series. We finish preparation by performing a Krakow suture with fiberwire on the tendon side (Fig. 3). Bone block, as stated, is drilled, and a guiding suture with an attached nitinol wire (Arthrex) is passed through it. This suture will be slided into the tibia and the nitinol guide will serve as a retroscrew driver guide (Fig. 4). This driver is specially designed for all inside fixation.

**Fig. 5:**
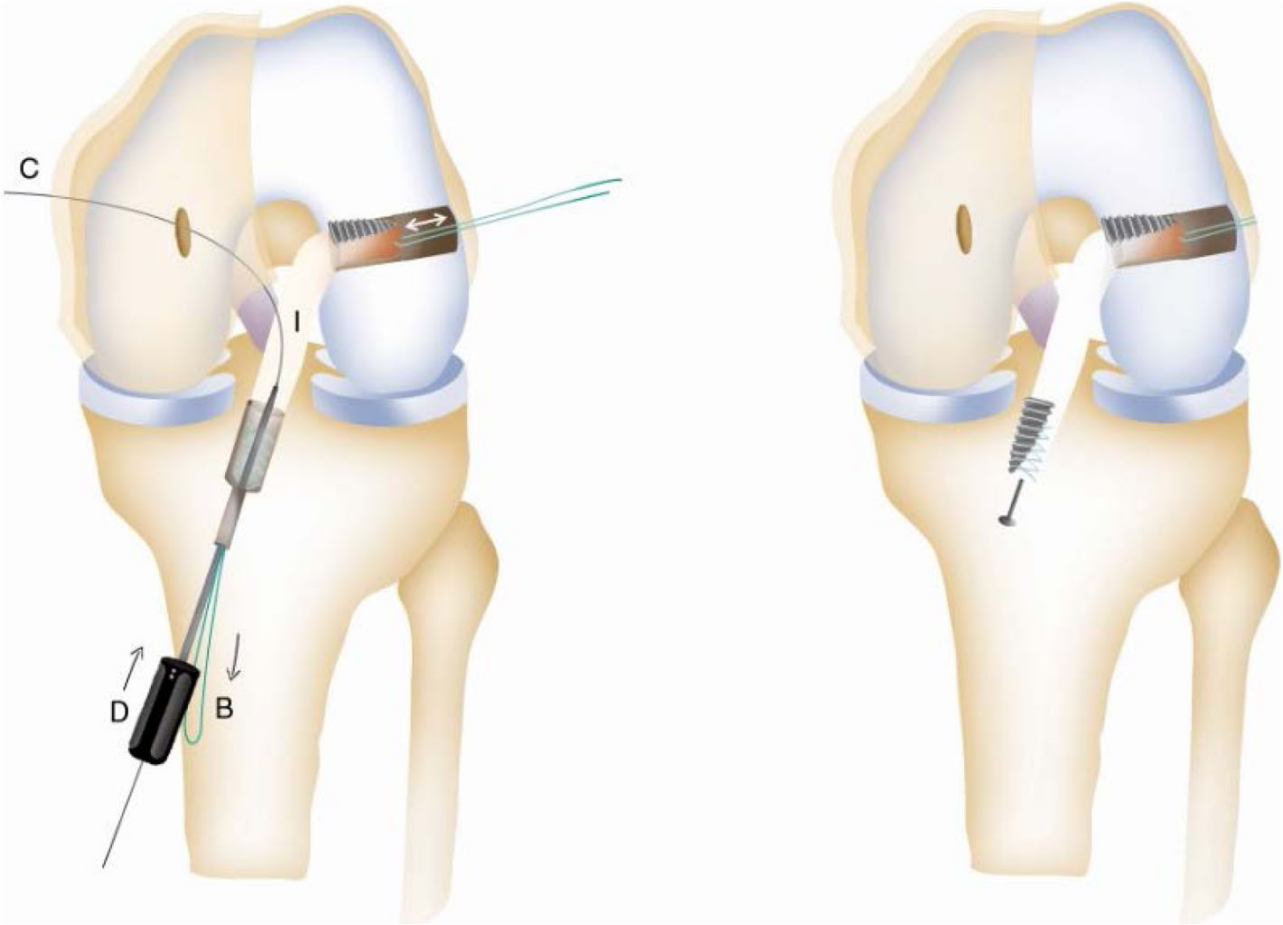
Graft fixation. 1- Sliding of the Retrodriver (D) on the nitinol wire (c) and femoral fixation with interference screw. 2- Tibial Fixation with Retroscrew. B. Krakow suture; C Nitinol eyeled wire; I QT Graft; D retrodriver.

Tibial ACL socket is performed with retrograde drilling, either with Retrodrill or Flipcutter on a Constant Guide (Arthrex). After introducing QT graft through low anteromedial portal, femoral fixation is performed first with an interference screw (tendon side) QT bone block is glided into tibial hole and fixed with a retroscrew, starting in 30 degrees flexion and finishing in full extension, as fixing screw in a retrograde fashion tends to tighten graft (Fig. 5).

### Rehabilitation

The initial goal is to reduce pain, inflammation and swelling, reestablish quadriceps control, and restore a normal gait. The knee is protected by a brace in the fully extended position for the first week, and full weight bearing is allowed. Quadriceps isometric exercises as well as straight-leg raising exercise, and passive range of motion, start as early as possible. Later series of closed kinetic-chain exercises are instructed. The range of motion should quickly recover to complete flexion and extension. Finally, aggressive quadriceps and hamstring muscles strengthening exercises are initiated.

Patients usually resume normal daily activities around 45 days after surgery, and typically return to sports activity after 7 months. Functional tests are performed at 3 and 6 months before allowing return to sports.

### Statistical analysis

The variability in functional scores was compared using the F test for equality of variances. *P* < .05 was considered statistically significant. All data are reported as mean standard deviation.

## Results (Fig. 6)

At 5 years follow up, Study group was composed of 291 patients who met the inclusion criteria. During January 2009 and December 2013, 548 ACL reconstructions with FQT and retrograde tibial fixation, using an all-inside technique were done. The following were excluded: 29 with Grade III/IV chondral lesions, 18 QT allografts, 22 patients with Multiligamentary reconstructions, 64 patients who could not be properly followed-up, 24 patients with revision surgery, and 100 patients in whom QT was harvested from the opposite knee. Out of 291, 268 (92.1%) were men and 23 (7.90%) women, 151 left knees and 140 right knees, average age was 23.2 years, (17–42 years) and average time to surgery was 45 days (15–467 days) (Table 1).

**Fig. 6:**
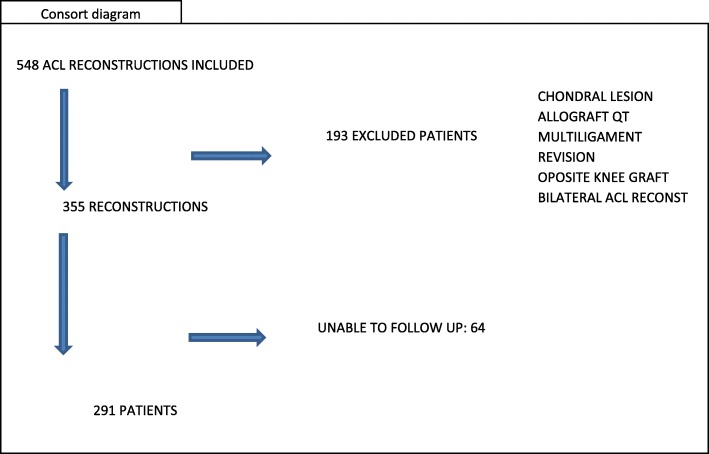
Consort Diagram. Inclusion and exclusion criteria

**Table 1:** Demographic table

Variables	N°	Average
Gender		
Male	268	92.09%
Female	23	7.9%
Side		
Left	151	51.89%
Right	140	48.1%
Age		
Min	17	23.2 Years
Max	42	
Time to surgery		
Min	15	45 Days
Max	467	

Average preop Lysholm score was 64 (SD:6.09). The average postop score at 5 years was 91 (SD:6.05). Concerning IKDC, previous to surgery, only 22% of patients were able to do moderate activity, while the remaining 78% could only lead a sedentary life. However, at 5 years postoperatively, 82% (*n* = 239) of patients were able to carry out high level physical activity, while only 10% were able to participate in mild activities.

At final evaluation, IKDC overall rating was A in 59.79% (*n* = 174) patients, B in 35.4% (*n* = 102), and C in 4.81% (*n* = 14).

Average time to return to sports was 8.2 months (6.5–11 months). Median pre-injury Tegner score was 9 (Range 4–10) while final median Tegner activity level at 5 years was 8 (Range 4–10) (*P* = 0.020). Only 73.3% of patients returned to their competitive pre-injury level.

After 5 years, stability measured by KT-1000 resulted in an average side-by-side difference of 1 mm +/− 1.3 mm. The percentage of patients with a difference less than or equal to 3 mm accounted for 89% of all the screened subjects; between 3 and 5 mm was 7%. In our series, only 4% of patients had a knee laxity measurement as high as 5 mm. Range of motion of the knee was normal in 87% of patients.

## Comorbidities and complications

At final follow-up visit, 5.15% (*n* = 15) of patients had anterior knee pain. Twelve out of these fifteen patients had patellofemoral syndrome-type pain, while only 3 had pain in the graft harvest site (quadriceps tendon tendinopathy). Additionally, 5 patients (1.71%) developed hematomas in the anterior aspect of the knee; these resolved spontaneously, without the need for surgical intervention. Only these patients underwent slightly milder rehabilitation in the first few weeks compared to the mean of patients.

Among complications, 2 patients had a patellar fracture at the time of harvesting the graft, which required fixation with consequent delay of the immediate postoperative motion; but none of these patients showed a decreased range of motion at the 5-year assessment. Besides, one of the patients had a late rupture of the quadriceps tendon, at 4 years after surgery, due to a new knee trauma.

It is also worth reporting a patient who developed an immediate postoperative MRSA infection requiring several sessions of surgical debridement.

Regarding ligament graft rerupture, at 5-year follow-up visit, the number of patients with ACL rerupture was 10.7% (*n* = 31).

## Discussion

The main findings of this study is that the use of FQT for ACL reconstruction showed a good functional outcome, as well as successful stability assessments, similar to those reported with other grafts such as BPTB [12, 21, 28, 40]. However, patients had less anterior knee pain. The outcome is also comparable to the results reported with the use of hamstrings for ACL reconstruction [6, 22], but with less flexor force deficit.

There are multiple reports on how to perform an ACL reconstruction with Quad Tendon: a) with bone block [2, 6, 7, 12, 13, 15, 19–22, 24–26, 28, 36, 41, 42] or b) without bone block [10, 23, 36]. Regarding fixation in cases with bone block, some authors prefer fixing the bone block to the femur [7, 26], while others, to the tibia [6, 41]. Most importantly, some authors prefer partial thickness [15, 21, 26] others full thickness [6, 41]. All these variables render assessment and comparison of results difficult. Ajay C. Kanakamedala et al. [17] conducted a systematic review in which no differences were found between the use of full-thickness versus partial-thickness quadriceps tendon; a result which is difficult to understand because in a hamstring graft, for instance, a 7 mm vs 10 mm thickness shows differences regarding rerupture risk [3]. This may be one of the explanations of the results regarding rerupture on the Danish Registry report [27] that does not differentiate between partial and full harvesting.

To our knowledge, the present report is one of the few that includes a large group of patients (291) with 5 year results, which is longer than the 48-month follow-up reported by Chen et al. [7], the 2, 8 years of Cavaignac et al. [6], or the 24 month minimum of Geib [11] or others. Furthermore, this is a young cohort of patients, younger than that reported by Chen et al. [7], which was 26 years old, and that reported by Kim et al. [21]. Another point to highlight is previous sporting level of the patients. In this study, patients who were operated had a higher level of sporting activity if compared to the rest of the publications such as Lee [26] (Tegner score of 4.7), or that of Cavaignac [6] (Tegner score of 7). Compared to other series, we obtained similar results in terms of Lysholm, IKDC, Lachman, percentage of tendinopathy and anterior knee pain.

The KT-1000 was used for the objective evaluation of the stability of the operated knee in relation to the non-operated knee. In this work we found that 89% of patients had a side-to-side difference of less than 3 mm; and that only 4% of the patients had a side-to-side difference of more than 5 mm. Results are similar to those described by Geib and Shelton [11], who reported 88.6% of patients with less than a 3 mm difference, and 5.3% of patients with a difference greater than 5 mm. Similar to reports from Kim et al. [21], Lund et al. [28] and Cavaignac [6].

Regarding IKDC, Chen reports [7] more patients in the normal group than this report (80% vs 59%), but with a shorter follow-up and without a description of the type of sports practiced. Our IKDC results are also in range with other studies.

We evaluated percentage of comorbidities, such as anterior knee pain, with the ability to walk on knees, as described by Kartus et al. [18]. We observed that, at the final follow-up visit, 5.15% of patients had anterior knee pain, but it did not prevent them from carrying out their daily life and sports activities. Same percentage is in the lower range reported in the literature [40]; these findings are probably influenced by the long follow-up period. It is important to emphasize the low incidence of this postoperative morbidity comparing to BPTB in literature. There are series of BPTB reconstructions that report up to 44% of anterior knee pain, and 48.1% of pain when kneeling [9, 21, 28, 40]. However, we reported a larger percentage of failures (10.7%) than the Danish report [27], probably the younger population (23 vs 28 years); very active (Postoperative Tegner Median 8) and the follow up (5 years vs 2 years), could explain this difference.

Something to keep in mind is that among patients undergoing revision, highest percentage of reruptures occurred in the tibia, where the tendon-bone block is fixed. This pattern of rerupture has been shown on biomechanical testing literature [37, 43]. As there are no other clinical reports in the literature about this subject, we cannot assure that this zone is the weak link, or that rerupture may be caused by use of a retrograde fixation or the placement of a bone block in the tibial zone.

One of the weaknesses of this study is the lack of a comparison group. Another weak point to take into account is the chronological time dispersion of patients from 2009 to 2014. In addition, there is a loss of (11.67%) follow-up, and the evaluation was not performed by an independent observer.

Although this “All Inside” FQT reports knee stability, complication rates and comorbidities, similar to other types of reconstructions technique, however our study is performed in a younger, more active sports involved population with a longer follow up. Nevertheless, we think that future prospective types comparing different quad reconstructions types are needed.
